# Long-Term Exposure to Traffic-Related Air Pollution and Risk of Incident Atrial Fibrillation: A Cohort Study

**DOI:** 10.1289/EHP392

**Published:** 2016-07-29

**Authors:** Maria Monrad, Ahmad Sajadieh, Jeppe Schultz Christensen, Matthias Ketzel, Ole Raaschou-Nielsen, Anne Tjønneland, Kim Overvad, Steffen Loft, Mette Sørensen

**Affiliations:** 1 Diet, Genes and Environment, Danish Cancer Society Research Center, Copenhagen, Denmark; 2 Department of Cardiology, Copenhagen University Hospital of Bispebjerg, Bispebjerg, Denmark; 3 Department of Environmental Science, Aarhus University, Roskilde, Denmark; 4 Section of Epidemiology, Department of Public Health, Aarhus University, Aarhus, Denmark; 5 Section of Environmental Health, Department of Public Health, Faculty of Health and Medical Sciences, University of Copenhagen, Copenhagen, Denmark

## Abstract

**Background:**

Atrial fibrillation is the most common sustained arrhythmia and is associated with cardiovascular morbidity and mortality. The few studies conducted on short-term effects of air pollution on episodes of atrial fibrillation indicate a positive association, though not consistently.

**Objectives:**

The aim of this study was to evaluate the long-term impact of traffic-related air pollution on incidence of atrial fibrillation in the general population.

**Methods:**

In the Danish Diet, Cancer, and Health cohort of 57,053 people 50–64 years old at enrollment in 1993–1997, we identified 2,700 cases of first-ever hospital admission for atrial fibrillation from enrollment to end of follow-up in 2011. For all cohort members, exposure to traffic-related air pollution assessed as nitrogen dioxide (NO_2_) and nitrogen oxides (NO_x_) was estimated at all present and past residential addresses from 1984 to 2011 using a validated dispersion model. We used Cox proportional hazard model to estimate associations between long-term residential exposure to NO_2_ and NO_x_ and risk of atrial fibrillation, after adjusting for lifestyle and socioeconomic position.

**Results:**

A 10 μg/m^3^ higher 10-year time-weighted mean exposure to NO_2_ preceding diagnosis was associated with an 8% higher risk of atrial fibrillation [incidence rate ratio: 1.08; 95% confidence interval (CI): 1.01, 1.14] in adjusted analysis. Though weaker, similar results were obtained for long-term residential exposure to NO_x_. We found no clear tendencies regarding effect modification of the association between NO_2_ and atrial fibrillation by sex, smoking, hypertension or myocardial infarction.

**Conclusion:**

We found long-term residential traffic-related air pollution to be associated with higher risk of atrial fibrillation. Accordingly, the present findings lend further support to the demand for abatement of air pollution.

**Citation:**

Monrad M, Sajadieh A, Christensen JS, Ketzel M, Raaschou-Nielsen O, Tjønneland A, Overvad K, Loft S, Sørensen M. 2017. Long-term exposure to traffic-related air pollution and risk of incident atrial fibrillation: a cohort study. Environ Health Perspect 125:422–427; http://dx.doi.org/10.1289/EHP392

## Introduction

Atrial fibrillation (AF) is the most common sustained arrhythmia and is associated with increased risk for ischemic and embolic stroke and myocardial infarction (Schnabel et al. 2015). Patients with symptomatic AF tend to experience reduced cardiac performance, exercise capacity, and quality of life (Go et al. 2001; Miyasaka et al. 2006). AF affects approximately 4% of the adult population ≥ 50 years old, but the etiology is complex and not fully understood (Go et al. 2001).

Exposure to short- and long-term ambient air pollution has been associated with cardiovascular morbidity and mortality, including myocardial infarction and stroke (Brook et al. 2010; Watkins et al. 2013). Only a few short-term studies have examined associations between ambient air pollution and an episode of AF with inconsistent results. No published study appears to have focused specifically on AF in relation to long-term exposure to air pollution. A few studies have investigated the association with mortality from cardiac rhythm disturbances. For example, one study found that residential particulate matter with an aerodynamic diameter ≤ 2.5 μm (PM_2.5_) assessed by ambient air monitors was associated with a higher risk (Pope et al. 2004), whereas two other studies failed to show any associations with exposure to PM_2.5_ and nitrogen dioxide (NO_2_) assessed by modelling (Beelen et al. 2009; Raaschou-Nielsen et al. 2012). Furthermore, another study examined the association between long-term air pollution and incident cardiac rhythm disturbances and found no associations with PM_10_ (particulate matter with an aerodynamic diameter ≤ 10 μm), NO_2_, or sulfur dioxide (SO_2_) assessed by emission-based models (Atkinson et al. 2013).

Exposure to air pollution might lead to AF through different mechanisms. Deposition of PM in the lung, with or without translocation into the bloodstream, has in controlled human exposure studies been found to induce local and systemic inflammation (Behbod et al. 2013; Graff et al. 2009; Kajbafzadeh et al. 2015). Systemic inflammation has in various studies been associated with an increased risk of AF (Dewland et al. 2015; Issac et al. 2007). Moreover, exposure to PM has been found to directly affect sensory receptors in the lung leading to alterations in the autonomic nervous system (Perez et al. 2015), which may induce changes of atrial electrophysiology and thereby play an important role in the initiation of AF (Chen et al. 2014; Shen and Zipes 2014). Furthermore, dysfunction of the autonomic nervous system can enhance inflammation (Tracey 2002). Besides these mechanisms, exposure to PM is known to cause oxidative stress which is suspected of inducing AF (e.g., through increasing inflammation and by damaging myofibrils), thereby potentially remodelling atrial structure (Miller et al. 2012; Youn et al. 2013). The small size of traffic-related PM, especially ultrafine particles from diesel engine emission, have a high alveolar deposition, large surface area and adhered toxic compounds including reactive oxygen species, and may therefore be particularly harmful in these mechanisms (Miller et al. 2012).

The aim of the present study was to investigate the association between residential exposure to long-term traffic-related air pollution, using NO_2_ and nitrogen oxides **(**NO_x_) as indicators of air pollution from traffic including particulate matter, and risk of incident AF in a large cohort.

## Methods

### Study Population

The present study was based on the Danish Diet, Cancer, and Health study (Tjønneland et al. 2007). Between 1993 and 1997, individuals 50–64 years old and living in Copenhagen or Aarhus were invited to participate in the study. Of the 160,725 individuals who were eligible for the study, 57,053 (35%) persons agreed to participate in the cohort. The participants had to be born in Denmark with no history of cancer at the time of enrollment. At enrollment, each participant completed self-administered, interviewer-checked, lifestyle questionnaires covering smoking habits, diet, alcohol consumption, physical activity, and education. The participants reported their current and past smoking habits of cigarettes, cheroots, cigars, and pipes. The quantity of tobacco smoked each day (smoking intensity) was calculated by converting the amount of each kind of tobacco into grams of tobacco by equating a cigarette to 1 g, a cheroot or a pipe to 3 g, and a cigar to 5 g of tobacco. Also, height, weight, waist circumference, and systolic and diastolic blood pressure were measured by trained staff members according to standardized protocols (Tjønneland et al. 2007). The study was conducted in accordance with the Helsinki Declaration and approved by the local Ethics Committees and written informed consent was obtained from all participants.

### Outcome Definition

Cases with AF diagnosed between baseline and death, emigration, or end of follow-up (31 December 2011) were identified by linking the unique personal identification number of each cohort member to the nationwide Danish National Patient Register (Lynge et al. 2011). Since 1977, patients with any disease diagnosed in the hospital are registered in the Danish National Patient Register and, since 1995, diagnoses from emergency rooms and outpatient visits have been registered as well (Lynge et al. 2011). Cases were identified using discharge diagnosis according to the *International Classification of Diseases* (ICD) 8 (codes 427.93 and 427.94) (WHO 1965) and ICD 10 (code I48.9) (WHO 1992). We considered only the first hospitalization of AF. In Denmark, all patients discharged from a hospital, an emergency room, or an outpatient clinic are registered into the National Patient Registry using the ICD10 (ICD8 before 1995). According to Danish and European guidelines, all patients with AF are ultimately referred to the hospital system for echocardiography and cardiology evaluation.

### Exposure Assessment

Residential address history for all cohort members between 1 January 1984 and date of AF event or end of follow-up at 31 December 2011 was collected from the Danish Civil Registration System (Pedersen 2011). Concentrations of ambient NO_2_ and NO_x_ were calculated with the Danish AirGIS (http://envs.au.dk/en/knowledge/air/models/airgis/) modelling system (dispersion model), for each year (1984–2011) at each address at which the cohort members had lived. NO_2_ and NO_x_ were used as indicators for air pollution, as they are considered good markers of traffic-related air pollution, and NO_x_ correlates closely with PM in Danish streets: *r* = 0.93 for total particle number concentration [size 10–700 nm (ultrafine particles)] and *r* = 0.70 for PM_10_ (Hertel et al. 2001; Ketzel et al. 2003). The Danish AirGIS modelling system calculates air pollution as the sum of regional background, urban background, and local street level calculated with the Operational Street Pollution Model (OSPM) (Berkowicz et al. 2008; Jensen et al. 2001; Kakosimos et al. 2010). Urban background concentration is calculated in a 1 km × 1 km resolution taking into account emissions from all sectors provided in the same spatial resolution, while the local street contribution is calculated at the building façade near the closest and most trafficked road with a few meters resolution. Input data for the AirGIS system included geographical coordinates for each address; road links with information on annual average daily traffic (average daily traffic on a roadway link during a period of 1 year expressed in vehicles per day), vehicle distribution (of light and heavy vehicles), travel speed, road type, emission factors for the Danish car fleet all including historic trends, street and building geometry, building height, and meteorological data. We obtained traffic counts for all Danish roads with more than 1,000 vehicles per day from a national road and traffic database (GIS-baseret national vej- og trafikdatabase 1960-2005) that includes an estimate about the historic changes in traffic back to 1960 (Jensen et al. 2009a). The AirGIS system has been successfully validated in various studies (Berkowicz et al. 2008; Jensen et al. 2009b; Ketzel et al. 2011). A validation study comparing modelled and measured NO_2_ has been conducted. The NO_2_ concentrations were measured in the years 1994–1995 using samplers placed at 204 positions in the greater Copenhagen area. At each location NO_2_ concentrations were measured during a consecutive period of 6 months with a sampling period of 1 month. The comparison between modelled and measured half-year mean NO_2_ concentrations showed a correlation coefficient (*R* ^2^) of 0.81, which on average was 11% lower than those modelled (Berkowicz et al. 2008). Also, modelled and measured 1-month mean concentrations of NO_x_ and NO_2_ over 12 years (1995–2006) on a busy street in Copenhagen (Jagtvej, 25,000 vehicles per day, street canyon) was correlated with correlation coefficients of 0.88 for NO_x_ and 0.67 for NO_2_. The modelled mean concentration over the whole 12-year period was 6% lower than the measured concentrations of NO_x_ and 12% lower than those of NO_2_ (Ketzel et al. 2011). Thus, the model predicted both geographical and temporal variation well.

### Statistical Analyses

The analyses were based on a Cox proportional hazards model with age as underlying time-scale (Thiébaut and Bénichou 2004). Left truncation at age of enrollment was used so that people were considered at risk from their exact age on the day they were enrolled into the cohort (delayed entry). All participants with a diagnosis of AF before enrollment were excluded. Right censoring was used at the age of AF (event), death, emigration, or end of follow-up (31 December 2011), whichever came first. Exposure to NO_2_ and NO_x_ were modelled as time-weighted mean air pollution for the 1, 5, and 10 years preceding diagnosis while taking all present and past addresses in these periods into account. The exposure windows (1, 5, and 10 years) were entered as time-dependent variables into the statistical risk model; thus, for each case of AF, we recalculated exposure for all cohort participants (at risk) at exactly the same age as the case at the time of diagnosis. To control for trends over time in outcome and exposure, we adjusted for calendar-year, which was estimated as an underlying time-dependent variable in 5-year intervals (1992–1997, 1997–2002, 2002–2007, and 2007–2012).

Incidence rate ratios (IRR) for the association between the two air pollution estimates and AF were analyzed crudely and adjusted for potential baseline confounders defined *a priori*: sex, body mass index (BMI; kilograms per meter squared), waist circumference (centimetres), smoking status (never, former, current), smoking duration (years), smoking intensity (lifetime average, gram tobacco/day), alcohol consumption (yes, no), intake of alcohol (gram/day), physical activity (yes, no), sport during leisure time (hours/week), length of school attendance (≤ 7, 8–10, > 10 years), and area-level socioeconomic position of the participant’s enrollment municipality (or district for Copenhagen; 10 districts in total) classified as low, medium, or high based on the municipality and district level, information on education, work market affiliation (yes or no) and income, occupational status (employed, retired or unemployed), and calendar-year. In an additional analysis, we included area-level socioeconomic position as random effect in the Cox model.

We also performed analyses with five categories of 10-year time-weighted exposure to NO_2_ and NO_x_ according to quintiles among cases and estimated IRR of AF using the lowest quintile as reference.

The assumption of linearity of NO_2_, NO_x_ and continuous covariates were evaluated visually and by formal testing (Wald’s test) with linear spline models with nine knots placed at deciles for cases. We found NO_x_ to deviate from linearity (*p* = 0.03), and therefore associations between NO_x_ and AF were analyzed using categories of NO_x_ (quintiles). NO_2_ did not deviate from linearity (*p* = 0.10), and associations between NO_2_ and AF were therefore analyzed both categorically (quintiles) and linearly (per 10 μg/m^3^). We also found smoking intensity to deviate from linearity and included this covariate as a spline with cut point at 20 g/day. This cut point was determined by visual examination of the linear spline model, followed by a formal testing of whether we could assume linearity of the variable below and above the cut point. The remaining covariates did not deviate from linearity.

We estimated whether there was an effect modification of the association between NO_2_ and AF according to the following variables: sex (men/women), smoking status (ever/never), area-level socioeconomic position (low/medium/high), baseline hypertension (yes/no), diagnosis of myocardial infarction (before censoring; yes/no), and a diagnosis of diabetes (before censoring though only until 2006; yes/no). Potential modification was evaluated by introducing an interaction term into the model and tested using the Wald test. Baseline hypertension was defined as having systolic blood pressure > 140 mm Hg or diastolic pressure > 90 mm Hg, measured by an automatic blood pressure device (Takeda Medical UA 751 or UA-743 oscillometric blood pressure and pulse rate monitor) at enrollment or answering yes to the following question in the baseline questionnaire: “Do you suffer, or have you ever suffered from high blood pressure?” Information on diagnosis of myocardial infarction before censoring was obtained by linking the unique personal identification numbers of all cohort members with the Danish National Patient Registry using ICD-8 (code 410) and ICD-10 (code DI21). We also analyzed whether the association between NO_2_ and AF diagnosed before 67.5 years old was different from the association between NO_2_ and AF diagnosed after the age of 67.5 years. We identified persons with diabetes by linking with the Danish National Diabetes Registry (Carstensen et al. 2008), for which we had information until 2006. All analyses were performed using SAS PROC PHREG (version 9.3; SAS Institute Inc.).

## Results

Of the 57,053 enrolled cohort participants, we excluded 572 participants with a diagnosis of cancer before enrollment, 1,015 participants with a diagnosis of AF before enrollment, 2,719 participants with incomplete residential history in the period from 1 January 1984 to censoring, and 2,276 participants with missing data on one or more covariates, leaving a study population of 50,399 participants. Among these, 2,700 participants were diagnosed with incident AF, during a mean follow-up time of 14.7 years.

The baseline median exposure to NO_2_ for the whole cohort was 16.6 μg/m^3^, ranging from the lowest value of 8.5 μg/m^3^ to a maximum of 65.3 μg/m^3^. For NO_x_ the baseline median exposure was 20.8 μg/m^3^ ranging from 10.3 μg/m^3^ to 379.6 μg/m^3^. The Spearman correlation between mean time-weighted 10-year exposure (preceding enrollment) to NO_2_ and NO_x_ was 0.97. The three exposure windows, 1-, 5-, and 10-year time-weighted mean were highly correlated, with Spearman’s correlation coefficients of > 0.90 for NO_2_ and 0.89 for NO_x_.

Distribution of baseline covariates among cohort participants according to low, medium and high NO_2_ exposure can be seen in Table 1. We observed that subjects living at residences with medium and high exposure to NO_2_ tended to have a higher socioeconomic position, to more often be heavy smokers and drinkers, and to have a higher occurrence of hypertension and history of myocardial infarction compared with participants with low-NO_2_ exposure. Fewer participants living in areas with high NO_2_ tended to be physically active compared with participants living in areas with low and medium NO_2_ exposure (Table 1).

**Table 1:** Baseline characteristics for the Diet, Cancer, and Health cohort according to low (NO_2_ < 15 μg/m^3^), medium (15 ≤ NO_2_ < 20 μg/m^3^), and high (NO_2_ ≥ 20 μg/m^3^) exposure to NO_2_ at enrollment of 50,399 cohort participants.

Characteristic at enrollment	Total cohort (*n* = 50,399)	NO_2_ < 15 μg/m^3^ (*n *= 15,652)	15 ≤ NO_2_ < 20 μg/m^3^ (*n* = 18,432)	NO_2_ ≥ 20 μg/m^3^ (*n* = 16,315)
Men (%)	47.0	48.9	46.6	45.5
Age (years)	56.2 (50.8–64.2)	55.8 (50.7–64.0)	56.5 (50.8–64.3)	56.3 (50.7–64.2)
Length of school attendance (%)
≤ 7 years	33.1	34.0	33.2	32.3
8–10 years	46.4	45.4	46.8	47.0
≥ 10 years	20.5	20.6	20.1	20.8
Socioeconomic position (%)^*a*^
Low	21.0	27.8	17.6	18.5
Medium	64.7	64.7	65.9	63.5
High	14.2	7.6	16.5	18.0
Smoking status (%)
Never	36.1	37.9	36.9	33.5
Former	36.4	28.7	28.2	25.6
Current	27.5	33.4	34.9	40.9
Among present and former smokers
Smoking duration (years)	33.0 (7.0–46.0)	35.0 (7.0–46.0)	32.0 (7.0–46.0)	33.0 (3.0–46.0)
Smoking intensity (g/day)^*b*^	14.7 (3.8–34.3)	14.5 (3.7–35.3)	14.6 (3.7–33.8)	15.0 (4.0–33.9)
Alcohol consumption (%)	97.8	98.2	97.7	97.4
Alcohol intake (g/day)	13.2 (1.1–64.4)	12.8 (1.2–59.7)	13.2 (1.1–64.6)	13.7 (1.0–69.2)
Physically active (%)	54.2	56.6	54.8	51.2
Sport during leisure time (hr/week)^*c*^	2.0 (0.5–7.0)	2.0 (0.5–7.0)	2.0 (0.5–6.5)	2.0 (0.5–7.0)
BMI (kg/m^2^)	25.5 (20.4–33.3)	25.5 (20.5–33.0)	25.6 (20.5–33.5)	25.6 (20.3–33.5)
Waist circumference (cm)	88.0 (69.0–110.0)	89.0 (69.5–109.5)	89.0 (69.0–110.0)	88.0 (69.0–110.0)
Hypertension (%)	49.9	48.2	50.6	50.7
History of myocardial infarction (%)	5.9	5.7	5.9	6.0
Note: Values are medians (5th–95th percentiles) unless otherwise stated. BMI, body mass index. ^***a***^Area-level socioeconomic position based on municipality information on education, work market affiliation and income. ^***b***^The average amount of tobacco smoked per day during lifetime. ^***c***^Among physically active adults.				

A 10-μg/m^3^ higher 10-year time-weighted mean exposure to NO_2_ was associated with an 8% (IRR = 1.08; 95% CI: 1.01, 1.14) higher risk of AF after adjustment (Table 2). The association between NO_2_ and AF seemed to follow a linear exposure–response relationship until around 18 μg/m^3^ with a possible levelling off at higher exposure levels, although the confidence intervals for each category were widely overlapping (see Figure S4). Including area-level socioeconomic position as a random effect resulted in only minor changes in the estimates (1.08; 95% CI: 1.02, 1.14). Exposure to NO_2_ for the two shorter exposure windows showed that a 10 μg/m^3^ increase in 1-year and 5-year time-weighted mean exposure preceding diagnosis was associated with a 7% (IRR = 1.07; 95% CI: 1.00, 1.13) and an 8% (IRR = 1.08; 95% CI: 1.02, 1.15) higher risk of AF, respectively (see Table S1 and Figure S2). A 10-year time-weighted mean exposure to more than 16.9 μg/m^3^ NO_x_ was associated with an increased risk of AF (Table 2). As found in the test for linearity, the association between exposure to NO_x_ and risk of AF deviated from linearity (see Figures S1 and S3).

**Table 2:** Association between mean 10-year residential exposure to NO_2_ and NO_x_ and risk of atrial fibrillation.

Air pollution	Cases	Crude IRR (95% CI)^*a*^	Adjusted IRR (95% CI)^*b*^
10-year mean NO_2_
Q1: < 11.4 μg/m^3^	540	1.00 (ref.)	1.00 (ref.)
Q2: 11.4–13.3 μg/m^3^	539	1.05 (0.93, 1.19)	1.06 (0.94, 1.20)
Q3: 13.3–16.1 μg/m^3^	541	1.10 (0.97, 1.24)	1.10 (0.97, 1.25)
Q4: 16.1–20.1 μg/m^3^	540	1.24 (1.10, 1.39)	1.24 (1.09, 1.40)
Q5: ≥ 20.1 μg/m^3^	540	1.19 (1.05, 1.34)	1.19 (1.04, 1.36)
Linear trend per 10 μg/m^3^^*c*^	2,700	1.08 (1.02, 1.15)	1.08 (1.01, 1.14)
10-year mean NO_x_
Q1: < 13.8 μg/m^3^	539	1.00 (ref.)	1.00 (ref.)
Q2: 13.8 < 16.9 μg/m^3^	540	0.99 (0.87, 1.11)	0.99 (0.87, 1.12)
Q3: 16.9 < 20.8 μg/m^3^	540	1.20 (1.06, 1.35)	1.20 (1.06, 1.36)
Q4: 20.8 < 29.6 μg/m^3^	541	1.20 (1.06, 1.35)	1.19 (1.05, 1.35)
Q5: ≥ 29.6 μg/m^3^	540	1.17 (1.03, 1.32)	1.16 (1.02, 1.32)
Note: CI, confidence interval; IRR, Incidence rate ratio; Q1–Q5, quintiles 1–5. ^***a***^Adjusted for age. ^***b***^Adjusted for age, sex, body mass index, waist circumference, smoking status, smoking duration, smoking intensity, intake of alcohol, sport during leisure time, length of school attendance, area-level socioeconomic position, and calendar year. ^***c***^Linear association per 10-μg/m^3^ increase in NO_2_ exposure.			

**Figure 1:**
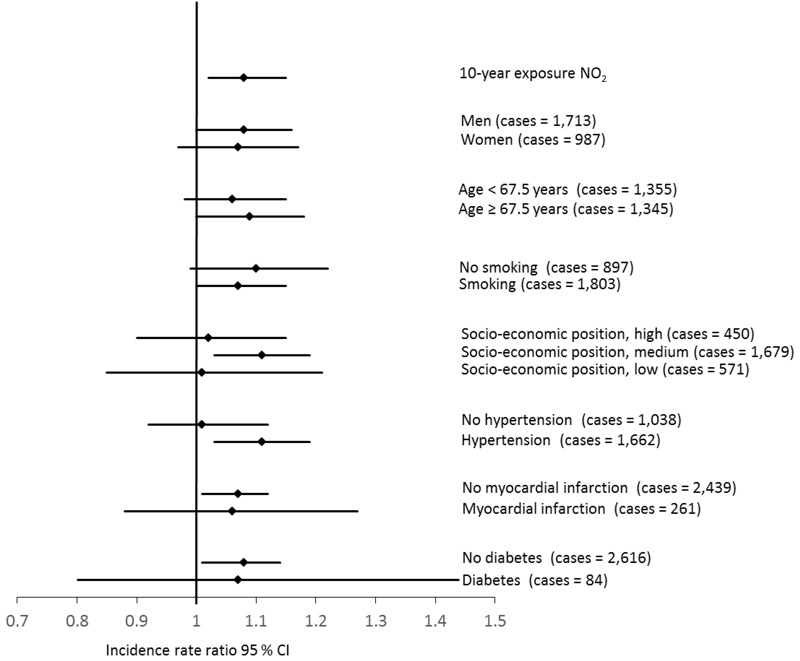
Associations (incidence rate ratios) between 10-year time-weighted exposure to NO_2_ (μg/m^3^) and risk for atrial fibrillation in different subgroups. Analyses were adjusted for age, sex, lifestyle factors (BMI, waist circumference, smoking status, smoking duration, smoking intensity, intake of alcohol, physical activity), socioeconomic position (length of school attendance, area-level socioeconomic position), and calendar year.

In Figure 1 associations between 10-year time-weighted exposures of NO_2_ and risk of AF in different subgroups are shown. There were no statistically significant differences in the association between exposure to NO_2_ and AF by sex, area-level socioeconomic position, smoking status, hypertension, myocardial infarction, or diabetes and no differences in estimates according to age at AF diagnosis. However, there was an indication of a stronger association between NO_2_ and AF among participants with a diagnosis of hypertension (IRR = 1.11; 95% CI: 1.04, 1.20) compared with no diagnosis of hypertension (IRR = 1.02; 95% CI: 0.92, 1.12).

## Discussion

We found a positive association between long-term residential exposure to traffic-related air pollution and risk of AF, which for NO_2_ seemed to follow a linear exposure–response relationship that leveled off after 18 μg/m^3^. There were no clear tendencies regarding effect modification by sex, smoking status, hypertension, or myocardial infarction in the association between air pollution and AF, and no differences in estimates according to age at AF diagnosis.

The present study is the first to specifically study associations between long-term air pollution and AF. Because of the absence of historical address data, most studies used short-term air-pollution assessed at the residential address at the time of AF. Five previous studies have investigated the association between short-term exposure to air pollution and episodes of AF (Bunch et al. 2011; Liao et al. 2011; Link et al. 2013; Milojevic et al. 2014; Rich et al. 2006). Although the studies are not consistent, most of them indicate that exposure to air pollution, over hours and days, is associated with increased risk of AF (Liao et al. 2011; Link et al. 2013; Milojevic et al. 2014; Rich et al. 2006), especially when NO_2_ and particles (PM_2.5_) are used as proxies for traffic-related air pollution. Although this is in accordance with the results of the present study, direct comparison is difficult as the mechanisms resulting in an episode of AF can differ from the mechanisms leading to the development of incident AF (Waks and Josephson 2014).

A few studies have investigated associations between long-term exposure to air pollution and cardiac rhythm disturbances; a broad group of arrhythmia diagnoses and different cardiac structural and functional abnormalities, which besides AF also includes attacks of tachycardia, ventricular fibrillation, cardiac arrest and others. The studies on mortality from cardiac rhythm disturbances show inconsistent results (Beelen et al. 2009; Pope et al. 2004; Raaschou-Nielsen et al. 2012). However, it is difficult to make a direct comparison between the present as well as short-term studies and these studies, as most cardiac rhythm disturbances do not result in death; in the present cohort, only 24 participants died of AF during the follow-up period. Only one study investigated incident cardiac rhythm disturbances as a health outcome (Atkinson et al. 2013). In contrast to the present study, Atkinson et al. (2013) found no associations between long-term exposure to air pollution assessed as PM_10_, NO_2_, and ozone by a dispersion model, and incidence of arrhythmias combined, although there was a significant positive association with SO_2_ (Atkinson et al. 2013). One reason for the discrepancy could be the differences in outcome definitions. In the present study, we only investigated risk of AF, which was based on data from the Danish National Patient Registry corresponding to approximately 60% of all the diagnoses included in arrhythmia outcome in the study by Atkinson et al. (2013). This could have diluted a potential association between air pollution and AF. Another reason could be the different approach used to estimate exposure to air pollution. In the study by Atkinson et al. (2013), the resolution was in 1 × 1 km grids and only one postcode for each cohort member. In the present study, we applied a dispersion model with much higher resolution to estimate exposure at the front door of each included address, taking into account street configurations and traffic data for the road links adjacent residence, and thus, potentially a more precise estimation of residential exposure to air pollution. In addition, the present study included exposure at both present and past addresses for all cohort members. The recruitment of the cohort for study and the outcome were also different in the two studies. We used a population-based approach with invitation of a random sample and used the highly complete Danish National Patient Register; whereas, Atkinson et al. (2013) recruited their cohort from 205 clinical practices in England.

We found that associations between NO_2_ and AF were not modified by sex, smoking status, hypertension, or myocardial infarction and no differences in estimates according to age at AF diagnosis. However, there were some indications of a stronger association among participants with hypertension at baseline, suggesting that hypertensive people are more susceptible to air pollution exposure in relation to developing AF. Hypertension is one of the strongest risk factors of AF and long-term hypertension is associated with abnormal atrial volume, structure, and function (Schnabel et al. 2009). These abnormalities may contribute to increased susceptibility of subjects with hypertension to air pollution. Interestingly, despite air pollution being a known risk factor of myocardial infarction (Wolf et al. 2015), which is an established risk factor of AF (Schnabel et al. 2015), the presence of a myocardial infarction before a diagnosis of AF did not modify the association between air pollution and AF. However, only about 10% of the cases had a diagnosis of myocardial infarction, and the event rate may therefore not be sufficient to detect a potential effect modification.

The associations observed between exposure to air pollution and risk of AF are relatively small compared to already established risk factors such as obesity and alcohol consumption (see Table S2) (Samokhvalov et al. 2010; Wanahita et al. 2008). However, even the relatively small association with AF observed may have substantial impact on the population given the widespread nature of air pollution.

The strengths of the present study include the prospective design with information on various potential lifestyle and socioeconomic confounders, the large number of cases, inclusion of only the first hospitalization of AF, and access to residential address history. Furthermore, cases with AF were identified using a high-quality nationwide hospital register, which has been found to have a very high positive predictive value with regard to the AF diagnosis (93%) (Rix et al. 2012).

The present study also has some limitations. Estimation of air pollution was based on a model, and although the AirGIS dispersion model is a standard, validated method, estimation of exposure is inevitably associated with some degree of uncertainty. As the exposure model does not distinguish between cases and noncase cohort members, such misclassification is likely to be non-differential. Also, we lacked information on factors that influence the personal exposure to air pollution, including time spent at home, information on commuting, and occupational exposure, which may result in exposure misclassification. Furthermore, we may have missed some AF cases as we were able only to include symptomatic AF, which led to hospital admission and outpatient visits. Therefore, the true incidence of AF is most likely higher than the observed incidence. However, in Denmark most patients with clinical symptoms of AF are referred to a hospital for further evaluation. Also, the date of diagnosis registered in the hospital register used might be somewhat different than the actual date of onset, which might underestimate the true effect (Lokken et al. 2009). Finally, we cannot rule out that residual confounding may be present. Although we adjusted for many risk factors for AF, adjustment resulted in only minor changes in the estimates, suggesting that residual confounding is not a major concern in the present study, though residual confounding by unmeasured characteristics is always a risk.

The present study is based on an urban cohort of elderly people and results may, therefore, not be readily generalizable to the general population.

## Conclusion

In conclusion, the study found a linear association between long-term exposure to traffic-related air pollution and risk of incident AF.

## Supplemental Material

(367 KB) PDF

## References

[r1] Atkinson RW, Carey IM, Kent AJ, van Staa TP, Anderson HR, Cook DG (2013). Long-term exposure to outdoor air pollution and incidence of cardiovascular diseases.. Epidemiology.

[r2] Beelen R, Hoek G, Houthuijs D, van den Brandt PA, Goldbohm RA, Fischer P (2009). The joint association of air pollution and noise from road traffic with cardiovascular mortality in a cohort study.. Occup Environ Med.

[r3] Behbod B, Urch B, Speck M, Scott JA, Liu L, Poon R (2013). Endotoxin in concentrated coarse and fine ambient particles induces acute systemic inflammation in controlled human exposures.. Occup Environ Med.

[r4] Berkowicz R, Ketzel M, Jensen SS, Hvidberg M, Raaschou-Nielsen O (2008). Evaluation and application of OSPM for traffic pollution assessment for a large number of street locations.. Environ Model Softw.

[r5] Brook RD, Rajagopalan S, Pope CA, Brook JR, Bhatnagar A, Diez-Roux AV (2010). Particulate matter air pollution and cardiovascular disease: an update to the scientific statement from the American Heart Association.. Circulation.

[r6] Bunch TJ, Horne BD, Asirvatham SJ, Day JD, Crandall BG, Weiss JP (2011). Atrial fibrillation hospitalization is not increased with short-term elevations in exposure to fine particulate air pollution.. Pacing Clin Electrophysiol.

[r7] Carstensen B, Kristensen JK, Ottosen P, Borch-Johnsen K, Steering Group of the National Diabetes Register (2008). The Danish National Diabetes Register: trends in incidence, prevalence and mortality.. Diabetologia.

[r8] Chen PS, Chen LS, Fishbein MC, Lin SF, Nattel S (2014). Role of the autonomic nervous system in atrial fibrillation: pathophysiology and therapy.. Circ Res.

[r9] Dewland TA, Vittinghoff E, Harris TB, Magnani JW, Liu Y, Hsu FC (2015). Inflammation as a mediator of the association between race and atrial fibrillation: results from the Health, Aging, and Body Composition Study.. JACC Clin Electrophysiol.

[r10] Go AS, Hylek EM, Phillips KA, Chang Y, Henault LE, Selby JV (2001). Prevalence of diagnosed atrial fibrillation in adults: national implications for rhythm management and stroke prevention: the AnTicoagulation and Risk Factors in Atrial Fibrillation (ATRIA) Study.. JAMA.

[r11] Graff DW Cascio WE Rappold A Zhou H Huang YCT Devlin RB 2009 Exposure to concentrated coarse air pollution particles causes mild cardiopulmonary effects in healthy young adults. Environ Health Perspect 117 1089 1094, doi:10.1289/ehp0900558 19654918 PMC2717135

[r12] Hertel O, Jensen SS, Andersen HV, Palmgren F, Wåhlin P, Skov H (2001). Human exposure to traffic pollution. Experience from Danish studies.. Pure Appl Chem.

[r13] Issac TT, Dokainish H, Lakkis NM (2007). Role of inflammation in initiation and perpetuation of atrial fibrillation: a systematic review of the published data.. J Am Coll Cardiol.

[r14] Jensen SS, Berkowicz R, Hansen HS, Hertel O (2001). A Danish decision-support GIS tool for management of urban air quality and human exposures.. Transp Res D Transp Environ.

[r15] Jensen SS, Hvidberg M, Pedersen J, Storm L, Stausgaard L, Becker T, et al (2009a). GIS-based National Street and Traffic Data Base 1960–2005 [in Danish]. National Environmental Research Institute (NERI) Technical Report 678.. http://www2.dmu.dk/Pub/FR678.pdf.

[r16] Jensen SS, Larson T, Deepti KC, Kaufman JD (2009b). Modeling traffic air pollution in street canyons in New York City for intra-urban exposure assessment in the US Multi-Ethnic Study of Atherosclerosis and Air Pollution.. Atmos Environ.

[r17] Kajbafzadeh M, Brauer M, Karlen B, Carlsten C, van Eeden S, Allen RW (2015). The impacts of traffic-related and woodsmoke particulate matter on measures of cardiovascular health: a HEPA filter intervention study.. Occup Environ Med.

[r18] Kakosimos KE, Hertel O, Ketzel M, Berkowicz R (2010). Operational Street Pollution Model (OSPM) – a review of performed validation studies, and future prospects.. Environ Chem.

[r19] Ketzel M, Berkowicz R, Hvidberg M, Jensen SS, Raaschou-Nielsen O (2011). Evaluation of AirGIS: a GIS-based air pollution and human exposure modelling system.. Int J Environ Pollution.

[r20] Ketzel M, Wåhlin P, Berkowicz R, Palmgren F (2003). Particle and trace gas emission factors under urban driving conditions in Copenhagen based on street and roof-level observations.. Atmos Environ.

[r21] Liao D, Shaffer ML, He F, Rodriguez-Colon S, Wu R, Whitsel EA (2011). Fine particulate air pollution is associated with higher vulnerability to atrial fibrillation—the APACR study.. J Toxicol Environ Health A.

[r22] Link MS, Luttmann-Gibson H, Schwartz J, Mittleman MA, Wessler B, Gold DR (2013). Acute exposure to air pollution triggers atrial fibrillation.. J Am Coll Cardiol.

[r23] Lokken RP, Wellenius GA, Coull BA, Burger MR, Schlaug G, Suh HH (2009). Air pollution and risk of stroke: underestimation of effect due to misclassification of time of event onset.. Epidemiology.

[r24] Lynge E, Sandegaard JL, Rebolj M (2011). The Danish National Patient Register.. Scand J Public Health.

[r25] Miller MR, Shaw CA, Langrish JP (2012). From particles to patients: oxidative stress and the cardiovascular effects of air pollution.. Future Cardiol.

[r26] Milojevic A, Wilkinson P, Armstrong B, Bhaskaran K, Smeeth L, Hajat S (2014). Short-term effects of air pollution on a range of cardiovascular events in England and Wales: case-crossover analysis of the MINAP database, hospital admissions and mortality.. Heart.

[r27] Miyasaka Y, Barnes ME, Gersh BJ, Cha SS, Bailey KR, Abhayaratna WP (2006). Secular trends in incidence of atrial fibrillation in Olmsted County, Minnesota, 1980 to 2000, and implications on the projections for future prevalence.. Circulation.

[r28] Pedersen CB (2011). The Danish Civil Registration System.. Scand J Public Health.

[r29] Perez CM, Hazari MS, Farraj AK (2015). Role of autonomic reflex arcs in cardiovascular responses to air pollution exposure.. Cardiovasc Toxicol.

[r30] Pope CA, Burnett RT, Thurston GD, Thun MJ, Calle EE, Krewski D (2004). Cardiovascular mortality and long-term exposure to particulate air pollution: epidemiological evidence of general pathophysiological pathways of disease.. Circulation.

[r31] Raaschou-Nielsen O Andersen ZJ Jensen SS Ketzel M Sørensen M Hansen J 2012 Traffic air pollution and mortality from cardiovascular disease and all causes: a Danish cohort study. Environ Health 11 60, doi:10.1186/1476-069X-11-60 22950554 PMC3515423

[r32] Rich DQ Mittleman MA Link MS Schwartz J Luttmann-Gibson H Catalano PJ 2006 Increased risk of paroxysmal atrial fibrillation episodes associated with acute increases in ambient air pollution. Environ Health Perspect 114 120 123, doi:10.1289/ehp.8371 16393668 PMC1332666

[r33] Rix TA, Riahi S, Overvad K, Lundbye-Christensen S, Schmidt EB, Joensen AM (2012). Validity of the diagnoses atrial fibrillation and atrial flutter in a Danish patient registry.. Scand Cardiovasc J.

[r34] Samokhvalov AV, Irving HM, Rehm J (2010). Alcohol consumption as a risk factor for atrial fibrillation: a systematic review and meta-analysis.. Eur J Cardiovasc Prev Rehabil.

[r35] Schnabel RB, Sullivan LM, Levy D, Pencina MJ, Massaro JM, D’Agostino RB (2009). Development of a risk score for atrial fibrillation (Framingham Heart Study): a community-based cohort study.. Lancet.

[r36] Schnabel RB, Yin X, Gona P, Larson MG, Beiser AS, McManus DD (2015). 50 year trends in atrial fibrillation prevalence, incidence, risk factors, and mortality in the Framingham Heart Study: a cohort study.. Lancet.

[r37] Shen MJ, Zipes DP (2014). Role of the autonomic nervous system in modulating cardiac arrhythmias.. Circ Res.

[r38] Thiébaut AC, Bénichou J (2004). Choice of time-scale in Cox’s model analysis of epidemiologic cohort data: a simulation study.. Stat Med.

[r39] Tjønneland A, Olsen A, Boll K, Stripp C, Christensen J, Engholm G (2007). Study design, exposure variables, and socioeconomic determinants of participation in Diet, Cancer and Health: a population-based prospective cohort study of 57,053 men and women in Denmark.. Scand J Public Health.

[r40] Tracey KJ (2002). The inflammatory reflex.. Nature.

[r41] Waks JW, Josephson ME (2014). Mechanisms of atrial fibrillation – reentry, rotors and reality.. Arrhythm Electrophysiol Rev.

[r42] Wanahita N, Messerli FH, Bangalore S, Gami AS, Somers VK, Steinberg JS (2008). Atrial fibrillation and obesity—results of a meta-analysis.. Am Heart J.

[r43] Watkins A, Danilewitz M, Kusha M, Massé S, Urch B, Quadros K (2013). Air pollution and arrhythmic risk: the smog is yet to clear.. Can J Cardiol.

[r44] WHO (World Health Organization) (1965). *International Classification of Diseases*. Eighth Revision.. http://sundhedsdatastyrelsen.dk/-/media/sds/filer/rammer-og-retningslinjer/klassisfikationer/sks-download/lukkede-klassifikationer/icd-8-klassifikation.txt?la=da.

[r45] WHO (1992). *International Statistical Classification of Diseases and Related Health Problems*. Tenth Revision.. http://apps.who.int/classifications/icd10/browse/2016/en.

[r46] Wolf K, Schneider A, Breitner S, Meisinger C, Heier M, Cyrys J (2015). Associations between short-term exposure to particulate matter and ultrafine particles and myocardial infarction in Augsburg, Germany.. Int J Hyg Environ Health.

[r47] Youn JY, Zhang J, Zhang Y, Chen H, Liu D, Ping P (2013). Oxidative stress in atrial fibrillation: an emerging role of NADPH oxidase.. J Mol Cell Cardiol.

