# Supplementary material for: Long-Term Exposure to Traffic-Related Air Pollution and Risk of Incident Atrial Fibrillation: A Cohort Study
Source: Environ Health Perspect. 2016 Jul 29;125(3):422–7. doi: 10.1289/EHP392 (PMC5332191; doi:10.1289/EHP392)
Supplement: (367 KB) PDF [file EHP392.s001.acco.pdf]

Environ Health Perspect
DOI: 10.1289/EHP392

Note to readers with disabilities: *EHP* strives to ensure that all journal content is accessible to all readers. However, some figures and Supplemental Material published in *EHP* articles may not conform to 508 standards due to the complexity of the information being presented. If you need assistance accessing journal content, please contact ehp508@niehs.nih.gov. Our staff will work with you to assess and meet your accessibility needs within 3 working days.

Supplemental Material

Long-Term Exposure to Traffic-Related Air Pollution and Risk of Incident Atrial Fibrillation: A Cohort Study

Maria Monrad, Ahmad Sajadieh, Jeppe Schultz Christensen, Matthias Ketzel, Ole Raaschou-Nielsen, Anne Tjønneland, Kim Overvad, Steffen Loft, and Mette Sørensen

Table of Contents

Table S1: Association between 1- and 5-years residential exposure to NO_2 and NO_x and risk of atrial fibrillation

Table S2: Associations between established risk factor and risk of atrial fibrillation in the Diet, Cancer and Health cohort.

Figure S1: Exposure-response relationship between 1-year time-weighted mean NO_x and risk of atrial fibrillation. The vertical whiskers show incidence rate ratios with 95 % confidence interval at the median of exposure categories (deciles).

Figure S2: Exposure-response relationship between 1-year time-weighted mean NO_2 and risk of atrial fibrillation. The vertical whiskers show incidence rate ratios with 95 % confidence interval at the median of exposure categories (deciles).

Figure S3: Exposure-response relationship between 10-years time-weighted mean NO_x and risk of atrial fibrillation. The vertical whiskers show incidence rate ratios with 95 % confidence interval at the median of exposure categories (deciles).

Figure S4: Exposure-response relationship between 10-years time-weighted mean NO_2 and risk of atrial fibrillation. The vertical whiskers show incidence rate ratios with 95 % confidence interval at the median of exposure categories (deciles).

Table S1: Association between 1- and 5-years residential exposure to NO_2 and NO_x and risk of atrial fibrillation

Air pollution	Cases	Crude IRR (95% CI) *	Adjusted IRR (95% CI) †
**1-year mean NO_2**			
Q1: < 10.3	538	1.00 (ref.)	1.00 (ref.)
Q2: 10.3- 11.8 μg/m^3	541	1.13 (1.00-1.28)	1.14 (1.01-1.29)
Q3: 11.8- 14.3 μg/m^3	541	1.15 (1.03-1.30)	1.16 (1.03-1.31)
Q4: 14.3- 18.1 μg/m^3	539	1.20 (1.06-1.35)	1.21 (1.06-1.37)
Q5: ≥ 18.1 μg/m^3	541	1.22 (1.08-1.38)	1.22 (1.07-1.39)
Linear trend per 10 μg/m^3‡	*2,700*	1.07 (1.01-1.14)	1.07 (1.00-1.13)
**5-year mean NO_2**			
Q1: < 10.9 μg/m^3	540	1.00 (ref.)	1.00 (ref.)
Q2: 10.9-12.4 μg/m^3	539	1.04 (0.93-1.18)	1.05 (0.93-1.18)
Q3: 12.4-15.2 μg/m^3	540	1.13 (1.00-1.27)	1.14 (1.01-1.29)
Q4: 15.2-19.0 μg/m^3	541	1.24 (1.10-1.39)	1.24 (1.10-1.40)
Q5: ≥ 19.0 μg/m^3	540	1.18 (1.05-1.34)	1.19 (1.05-1.36)
Linear trend per 10 μg/m^3‡	*2,700*	1.09 (1.03-1.15)	1.08 (1.02-1.15)
**1-year mean NOx**			
Q1: < 11.4 μg/m^3	539	1.00 (ref.)	1.00 (ref.)
Q2: 11.4-13.3 μg/m^3	541	1.14 (1.01-1.28)	1.15 (1.02-1.30)
Q3: 13.3-16.1 μg/m^3	539	1.18 (1.04-1.32)	1.19 (1.05-1.34)
Q4: 16.1-20.1 μg/m^3	541	1.21 (1.07-1.36)	1.22 (1.07-1.38)
Q5: ≥ 20.1 μg/m^3	540	1.23 (1.09-1.38)	1.23 (1.08-1.14)
**5-year mean NO_x**			
Q1: < 13.8 μg/m^3	539	1.00 (ref.)	1.00 (ref.)

Q2: 13.8 < 16.9 μg/m^3	541	1.06 (0.94-1.19)	1.06 (0.94-1.20)
Q3: 16.9 < 20.8 μg/m^3	539	1.22 (1.08-1.38)	1.24 (1.10-1.41)
Q4: 20.8 < 29.6 μg/m^3	540	1.19 (1.05-1.34)	1.19 (1.05-1.35)
Q5: ≥ 29.6 μg/m^3	541	1.23 (1.09-1.39)	1.23 (1.08-1.40)

IRR, Incidence rate ratio; CI, confidence interval; Q1-Q5, quintiles 1-5.
[*] Adjusted for age
†Adjusted for age, sex, body mass index, waist circumference, smoking status, smoking duration, smoking intensity, intake of alcohol, sport during leisure time, length of school attendance, area level socioeconomic position and calendar year.
‡ Linear association per 10 μg/m^3 increase in NO_2 exposure

Table S2: Associations between established risk factor and risk of atrial fibrillation in the Diet, Cancer and Health cohort.

Characteristic at enrolment	Per increase in interquartile range	IRR (95 % CI)
NO_2 *	6.76 μg/m^3	1.05 (1.00-1.09)
Sex		
Women		ref.
Men		2.13 (1.97-2.30)
Smoking status		
Never		ref.
Former		1.16 (1.06-1.28)
Current		1.17 (1.07-1.28)
Smoking duration†	18 years	1.07 (1.00-1.15)
Alcohol intake ‡	25.0 g/day	1.26 (1.22-1.31)
BMI	4.94 kg/m^2	1.39 (1.34-1.45)
Waist circumference (cm)	18 cm	1.83 (1.74-1.92)

IRR, Incidence rate ratio; CI, confidence interval
* Baseline NO_2
† Among present and former smokers
‡ Among drinkers

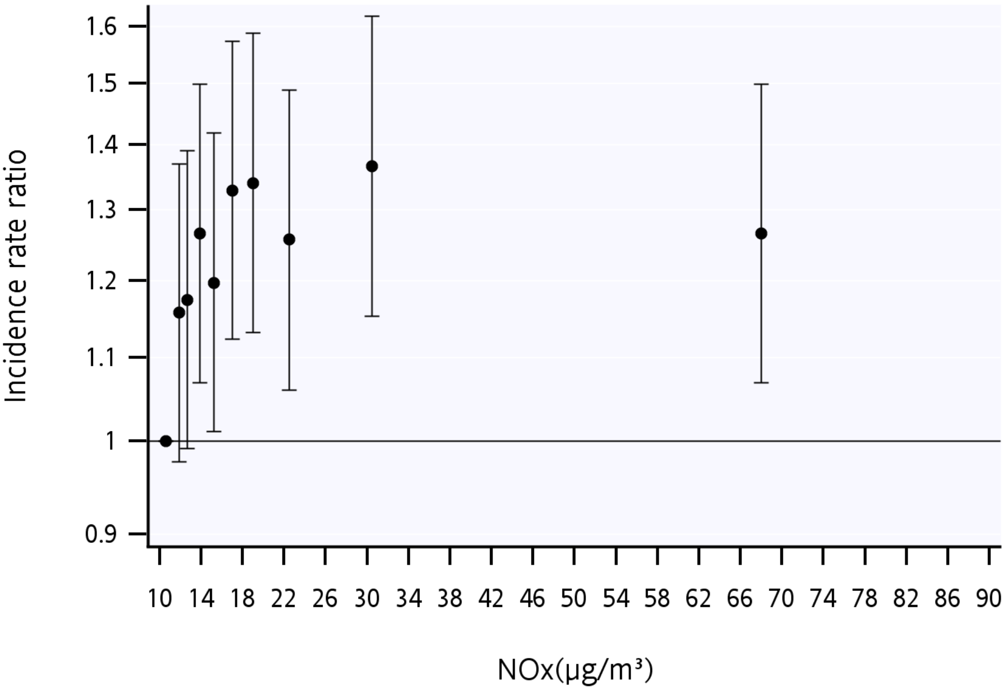

Figure S1: Exposure-response relationship between 1-year time-weighted mean NO_x and risk of atrial fibrillation. The vertical whiskers show incidence rate ratios with 95 % confidence interval at the median of exposure categories (deciles).

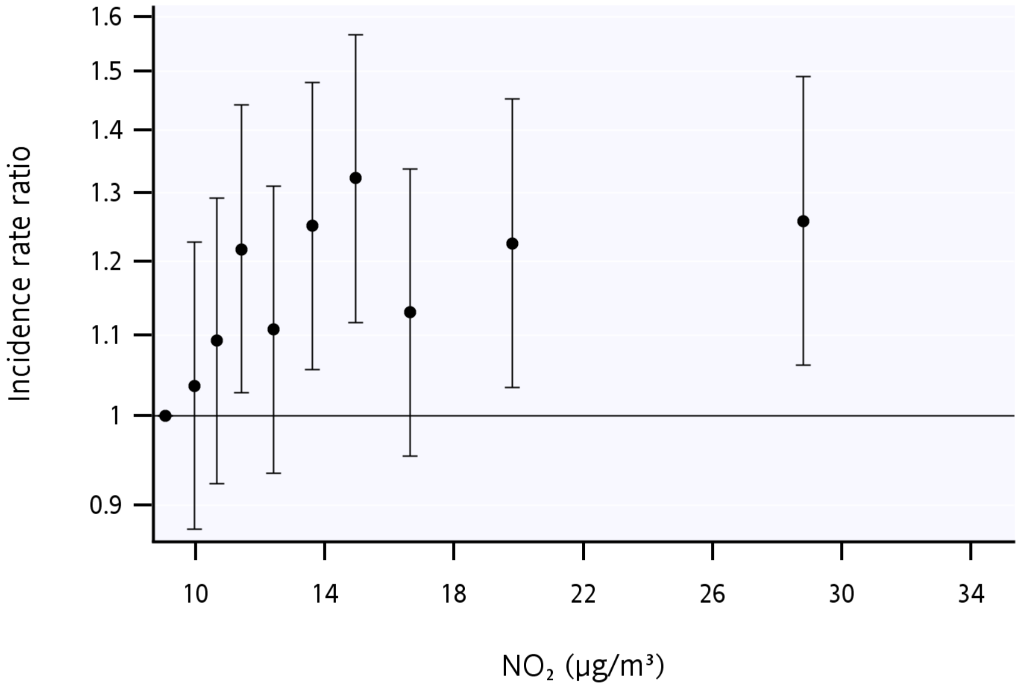

Figure S2: Exposure-response relationship between 1-year time-weighted mean NO_2 and risk of atrial fibrillation. The vertical whiskers show incidence rate ratios with 95 % confidence interval at the median of exposure categories (deciles).

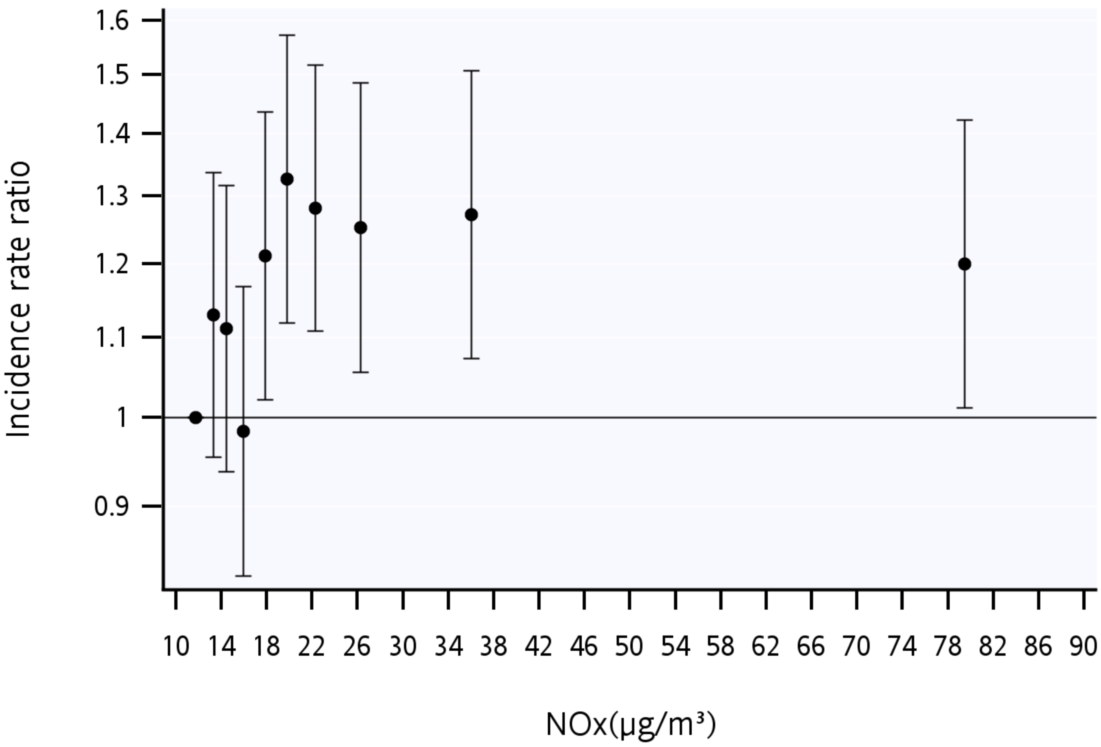

Figure S3: Exposure-response relationship between 10-years time-weighted mean NO_x and risk of atrial fibrillation. The vertical whiskers show incidence rate ratios with 95 % confidence interval at the median of exposure categories (deciles).

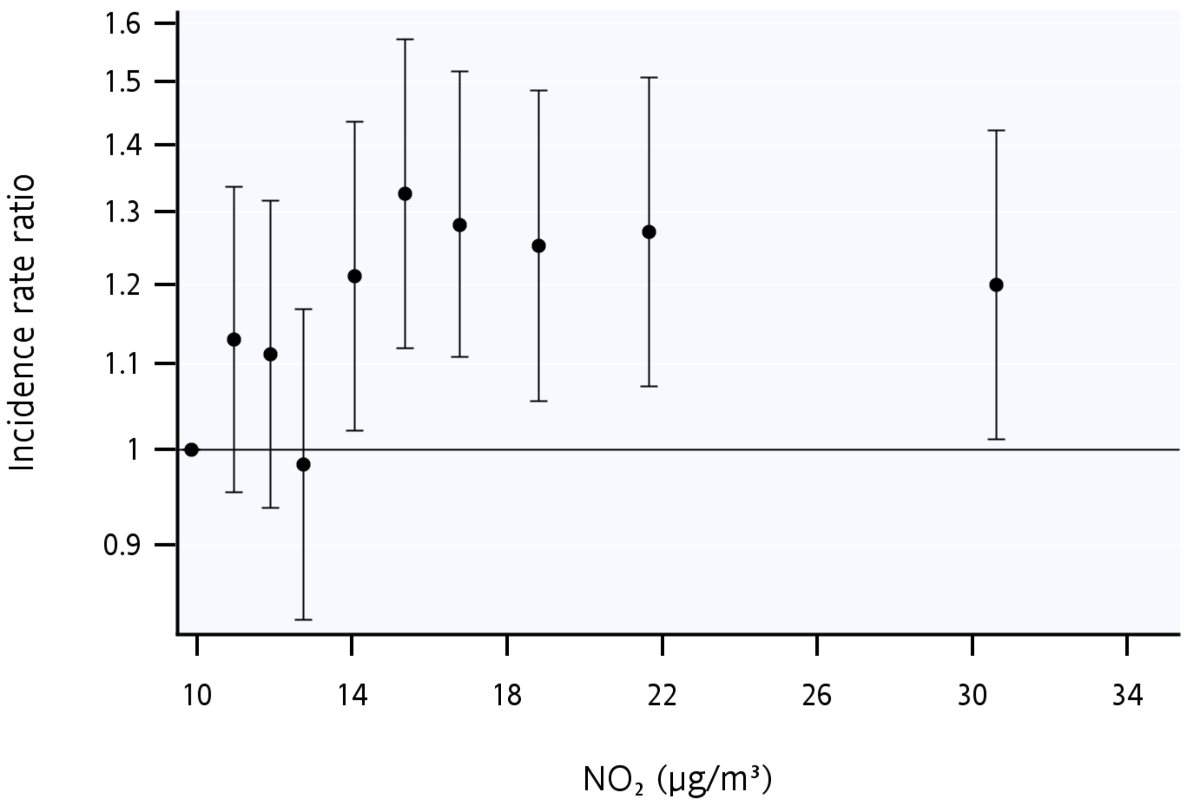

Figure S4: Exposure-response relationship between 10-years time-weighted mean NO_2 and risk of atrial fibrillation. The vertical whiskers show incidence rate ratios with 95 % confidence interval at the median of exposure categories (deciles).
